# Duplicated zebrafish (*Danio rerio*) inositol phosphatases *inpp5ka* and *inpp5kb* diverged in expression pattern and function

**DOI:** 10.1007/s00427-023-00703-z

**Published:** 2023-05-15

**Authors:** Dhyanam Shukla, Brian M. Gural, Edmund S. Cauley, Namarata Battula, Shorbon Mowla, Brittany F. Karas, Llion E. Roberts, Luca Cavallo, Luka Turkalj, Sally A. Moody, Laura E. Swan, M. Chiara Manzini

**Affiliations:** 1 Department of Neuroscience and Cell Biology and Child Health Institute of New Jersey, Rutgers Robert Wood Johnson Medical School, 89 French Street, CHINJ Rm 3274, New Brunswick, NJ 08901 USA; 2 Department of Biochemistry and Molecular Medicine, School of Medicine and Health Sciences, The George Washington University, Washington, DC USA; 3 Institute of Systems, Molecular and Integrative Biology, University of Liverpool, Liverpool, UK; 4 Department of Anatomy and Cell Biology, School of Medicine and Health Sciences, The George Washington University, Washington, DC USA

**Keywords:** Inositol phosphatase, INPP5K, Zebrafish, Gene duplication

## Abstract

One hurdle in the development of zebrafish models of human disease is the presence of multiple zebrafish orthologs resulting from whole genome duplication in teleosts. Mutations in inositol polyphosphate 5-phosphatase K (*INPP5K*) lead to a syndrome characterized by variable presentation of intellectual disability, brain abnormalities, cataracts, muscle disease, and short stature. INPP5K is a phosphatase acting at position 5 of phosphoinositides to control their homeostasis and is involved in insulin signaling, cytoskeletal regulation, and protein trafficking. Previously, our group and others have replicated the human phenotypes in zebrafish knockdown models by targeting both *INPP5K* orthologs *inpp5ka* and *inpp5kb*. Here, we show that *inpp5ka* is the more closely related orthologue to human *INPP5K.* While both *inpp5ka* and *inpp5kb* mRNA expression levels follow a similar trend in the developing head, eyes, and tail, *inpp5ka* is much more abundantly expressed in these tissues than *inpp5kb*. In situ hybridization revealed a similar trend, also showing unique localization of *inpp5kb* in the pineal gland and retina indicating different transcriptional regulation. We also found that *inpp5kb* has lost its catalytic activity against its preferred substrate, PtdIns(4,5)P_2_. Since most human mutations are missense changes disrupting phosphatase activity, we propose that loss of *inpp5ka* alone can be targeted to recapitulate the human presentation. In addition, we show that the function of *inpp5kb* has diverged from *inpp5ka* and may play a novel role in the zebrafish.

**Supplementary Information:**

The online version contains supplementary material available at 10.1007/s00427-023-00703-z.

## Introduction

Inositol polyphosphate 5-phosphatase K (INPP5K [MIM:607875]) is a highly conserved phosphatase that participates in the regulation of phosphoinositide (PI) signaling. Also referred to as skeletal muscle and kidney-enriched inositol phosphatase (*SKIP*), *INPP5K* is highly expressed in the brain, eyes, and muscles during development and adulthood (Ijuin et al. 2000; Gurung et al. 2003). In humans, homozygous or compound heterozygous mutations in *INPP5K* have been causally linked to a form of muscular dystrophy with cataracts and intellectual disability (MIM: 617404) also associated with short stature, and microcephaly with considerable variability in the age of onset and clinical presentation (Osborn et al. 2017; Wiessner et al. 2017; Yousaf et al. 2017; D’Amico et al. 2020; Hathazi et al. 2021). Similarities have been noted with Marinesco-Sjögren syndrome (MIM: 248800), a form of myopathy also associated with congenital cataracts, short stature, and cerebellar ataxia (Senderek et al. 2005; Krieger et al. 2013).

PIs are a category of lipid molecules that play crucial roles in signal transduction, ion channel regulation, cellular migration, membrane trafficking, vesicle transport, and many other processes (Di Paolo and De Camilli 2006; Balla 2013; Raghu et al. 2019). The seven unique members of this group are distinguished by their patterns of phosphorylation of the inositol head (PtdIns), which can occur at one or more of three positions (-3, -4, or -5). Production of PIs is regulated by an array of kinases and phosphatases (Balla 2013). INPP5K hydrolyzes the D-5 position of the inositol ring in both PtdIns(4,5)P_2_ and PtdIns(3,4,5)P_3_, with highest activity for PtdIns(4,5)P_2_ (Ijuin et al. 2000; Vandeput et al. 2006; Davies et al. 2015). INPP5K is largely localized to the endoplasmic reticulum (ER) (Gurung et al. 2003; Dong et al. 2018) but can translocate to membrane ruffles as part of a complex with the glucose-regulated protein GRP78/BiP to negatively regulate insulin receptor signaling via phosphatidylinositol-3-kinase (PI3K) (Ijuin and Takenawa 2003; Ijuin et al. 2015, 2016a, b).

Multiple zebrafish (*Danio rerio*) models of *INPP5K* loss of function have been generated using morpholino oligonucleotides (MOs) targeting both paralogs, *inpp5ka* and *inpp5kb* (Osborn et al. 2017; Wiessner et al. 2017; Hathazi et al. 2021). However, when the genes were targeted independently, knockdown of *inpp5ka* was sufficient to yield phenotypes typical of neurological and muscular disorders, such as microphthalmia, microcephaly, shortened body, reduced touch-evoked motility, and myopathy. In contrast, *inpp5kb* MOs produced a mild phenotype in a small subset of morphants (Osborn et al. 2017). In addition, we found *inpp5ka* expression to be significantly higher than *inpp5kb* in zebrafish embryos at 2 days post fertilization (dpf) (Osborn et al. 2017). These findings suggested that *inpp5ka* may be the most conserved human paralog and *inpp5kb* function may have diverged.

Due to a genome duplication event in teleost fish, about 30% of zebrafish genes have a paralog (Howe et al. 2013), but duplicated genes often acquire differential expression and function (Postlethwait et al. 1998; Ravi and Venkatesh 2018). In this study, we sought to better characterize expression patterns and function of *inpp5ka* and *inpp5kb* to understand whether they diverged and support the development of better models of *INPP5K* mutations in humans. We show that both *inpp5ka* and *inpp5kb* have a dynamic developmental expression in the eyes, head, and tail, but found that *inpp5kb* is expressed at lower levels and specifically enriched in the pineal gland and the inner nuclear layer of the retina. In addition, Inpp5kb lost the majority of its phosphatase activity for PtdIns(4,5)P_2_ which is the preferred substrate for INPP5K (Ijuin et al. 2000). Together, these data indicate that *inpp5ka* is the closest ortholog to *INPP5K* and suggest a unique role for *inpp5kb* within the zebrafish neural tissues.

## Methods

### Animal care

Maintenance and husbandry of zebrafish (*Danio rerio*) breeders and larvae were performed following protocols approved by the Institutional Animal Care and Use Committee of George Washington University and Rutgers University. All animals were from AB or EK backgrounds.

### Protein alignments

Clustal Omega and Jalview were used to align the sequences for all transcripts and define conservation (Sievers et al. 2011). Percent identities between the human INPP5K (NP_057616.2), zebrafish Inpp5ka (NP_001082962.2), and Inpp5kb (XP_021335021.1) were calculated using Jalview (Waterhouse et al. 2009).

### Quantitative PCR analysis

Samples were collected at 1, 2, 3, 4, 5, and 30 dpf and at 1-year-old for adults. Whole zebrafish embryos and larvae or micro-dissected tissue from eyes, head, and tails were pooled and RNA was extracted using the ReliaPrep RNA Miniprep System kit (Promega, Madison, WI). RNA was treated with DNase I (New England Biolabs) and complementary DNA (cDNA) was synthesized using the iScript cDNA Synthesis kit (Bio-Rad). 600 ng of cDNA per sample were analyzed via qPCR using the SsoFast EvaGreen Supermix (Bio-Rad) or Power UP SYBR Green Master Mix (Thermo Fisher). All reactions were run with 3 technical replicates and repeated on at least 3 biological replicates for 40 cycles on a QuantStudio 3 RealTime PCR system and recorded with QuantStudio Design and Analysis software. Custom primers were designed for *inpp5ka* (ex9_F: 5′-TGGGACTGGATTGGGTTAT-3′; ex10_R:5′-GCTCCTCATTGAAAGACACC-3′) and *inpp5kb* (ex2_F: 5′-CGACCACTGACCTCTATGTG-3′; ex3_R: 5′-ATGAGGAGGTGACTCCATGT-3′) and housekeeping controls elongation factor 1 alpha *eef1a* (eef1a_F: 5′GGGCAAGGGCTCCTTCAA-3′; eef1a_R: 5′-CGCTCGGCCTTCAGTTTG-3′) and riboprotein L18 *rpl18* (rpl18_F: 5′-GGCTAAGGTGATGTTTTCGTG-3′; rpl18_R: 5′-GCACATTGCCAATGTTCAGC-3′).

### Whole-mount in situ hybridization

Full-length *inpp5ka* (NM_001089493.1) and *inpp5kb* (XM_021479346.1) cDNAs were cloned into the pCS2 + plasmid (Addgene). Digoxygenin-labeled sense and antisense probes were synthesized from the linearized plasmids using the DIG RNA Labeling Kit (SP6/T7) (Roche/MilliporeSigma). Whole-mount ISH was performed as previously described (Yan et al. 2009). Expression patterns were confirmed in at least 5 independent embryos per probe and representative images are shown.

### RNA scope analysis

Zebrafish larvae were euthanized at 5 dpf by tricaine methanesulfonate and fixed in fresh 4% PFA for 24 h at 4 °C. Larvae were rinsed with 1 × PBS three times and cryoprotected with a 15% and 30% sucrose gradient with 0.025% sodium azide. The fish were embedded in tissue freeze medium (TFM) (General Data Healthcare) and frozen in 2-methyl butane with dry ice. Cryosections were performed using a Leica CM1850 UV Cryostat (Leica Microsystems) at 12 μm and mounted on SuperFrost Plus Slides (Fisher Scientific). The RNAscope assay was performed using the Multiplex Fluorescent Reagent Kit (Advanced Cell Diagnostics) according to the protocol provided for fixed-frozen tissue samples (UM 323,100). The following modifications were made: post fixation in 4% PFA at 4 °C was increased to 20 min, antigen retrieval was performed for 5 min using a microwave to help the target retrieval buffer reach boiling point, and RNAscope Protease Plus was used instead of RNAscope Protease III. The samples were hybridized using custom-designed RNAscope target probes for *inpp5ka* (Dr-inpp5ka-C1, Cat# 1,224,361-C1) and *inpp5kb* (Dr-inpp5kb-C2, Cat# 1,224,371-C2). In addition, two positive control genes were used for each target gene, *polr2a* (Dr-polr2a-C1) and *ppib* (Dr-ppib-C2). The negative control gene used in this experiment was DapB (DapB-C1,C2) expressed in *Bacillus subtilis*. We used the TSA Vivid Fluorophore 520 (Cat# 323,271) to develop the HRP-C1 signal and TS Vivid Fluorophore 650 (Cat# 323,273) to develop the HRP-C2 signal; both at a concentration of 1:750. Slides were coverslipped using ProLong™ Gold Antifade Mountant (Thermo Fisher). Images were taken using a Zeiss LSM800 confocal microscope and Zeiss Zen imaging software. Expression patterns were confirmed in at least 5 independent larvae per probe and representative images are shown.

### Phosphatase assay

Full-length *inpp5ka* (NM_001089493.1) and *inpp5kb* (XM_021479346.1) cDNAs were generated by gene synthesis and cloned into the pGEX-1 to generate GST-fusion proteins (Genewiz/Azenta Life Sciences). GST-human INPP5K and GST were used as positive and negative controls respectively (Weissner et al. 2017). Constructs were transformed into BL21 DE3 pLysS, induced with 100uM IPTG overnight, and harvested by centrifugation. Cells were lysed in assay buffer (50 mM Tris–HCl [pH 7.5], 150 mM NaCl, 10 mM MgCl_2_) plus 1% Triton X-100, EDTA-free protease inhibitors (Roche Diagnostics) and turbonuclease (Sigma). GST fusion proteins were affinity purified over gluthione sepharose 4B (GE Healthcare). After extensive washing, aliquots of beads were run on Coomassie gels to determine the abundance of full-length fusion proteins. Beads bearing equal amounts of fusion proteins were incubated in assay buffer containing 135 μM PtdIns(4,5)P_2_diC8 or PtdIns(3,4,5)P_3_diC8, including control wells with no enzyme or no substrate lipid, and incubated for 1 h at 37C. Free phosphate was measured using the Malachite Green assay kit (Echelon Biosciences). Results of three independent experiments were presented as mean ± standard deviation. To minimize variability between purifications, all constructs were freshly prepared and purified in parallel for each experiment.

## Results

### Zebrafish and human INPP5K protein alignments

**Fig. 1:**
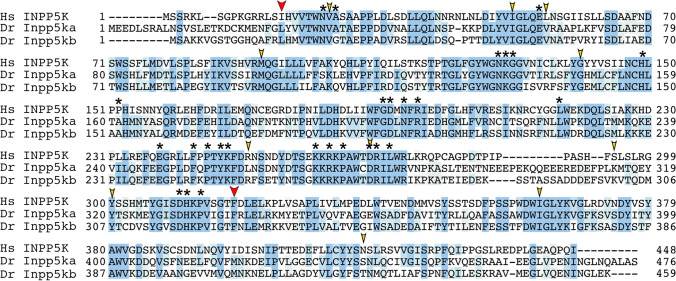
Protein alignment of INPP5K, Inpp5ka, and Inpp5kb highlighting conserved amino acids required for phosphatase activity. The start and end of the catalytic domain in the human protein are marked with large red arrowheads. Amino acids required for phosphatase activity have been denoted with asterisks (*). Small arrowheads indicate residues that are altered by missense variants in humans. Hs, *Homo sapiens*; Dr, *Danio rerio*

To determine whether *inpp5ka* and *inpp5kb* lead to functionally divergent proteins, we first analyzed their protein sequence. Protein sequence alignment of INPP5K (NP_057616.2), Inpp5ka (NP_001082962.2), and Inpp5kb (XP_021335021.1) revealed 45.8% and 43.9% identity between the human orthologue and Inpp5ka and Inpp5kb respectively, while the zebrafish proteins showed 62.2% identity with each other (Fig. 1). Higher conservation was present in the phosphatase domain with 49.7% amino acid sequence identity between the human protein and either zebrafish protein. It is important to note that while the primary isoforms listed above are the most similar to the human gene, both *inpp5ka* and *inpp5kb* have additional predicted transcripts that are not found in humans. One transcript has an alternative exon 1 (*inpp5ka*: XM_009291785.3 and *inpp5kb*: XM_021479345.1) adding an N-terminal sequence of 63 amino acids for Inpp5ka and 48 amino acids for Inpp5kb. A small 27 base pair alternative exon 9 was also identified in predicted transcripts both *inpp5ka* (XM_00557623.4) and *inpp5kb* (XM_005155275.4). All expression and functional analyses performed in this study were based on the sequence of the primary isoforms most similar to the human gene, but the design for the primer and probes did not exclude other predicted isoforms.

### Divergent expression and localization of INPP5K orthologs in zebrafish larva

**Fig. 2:**
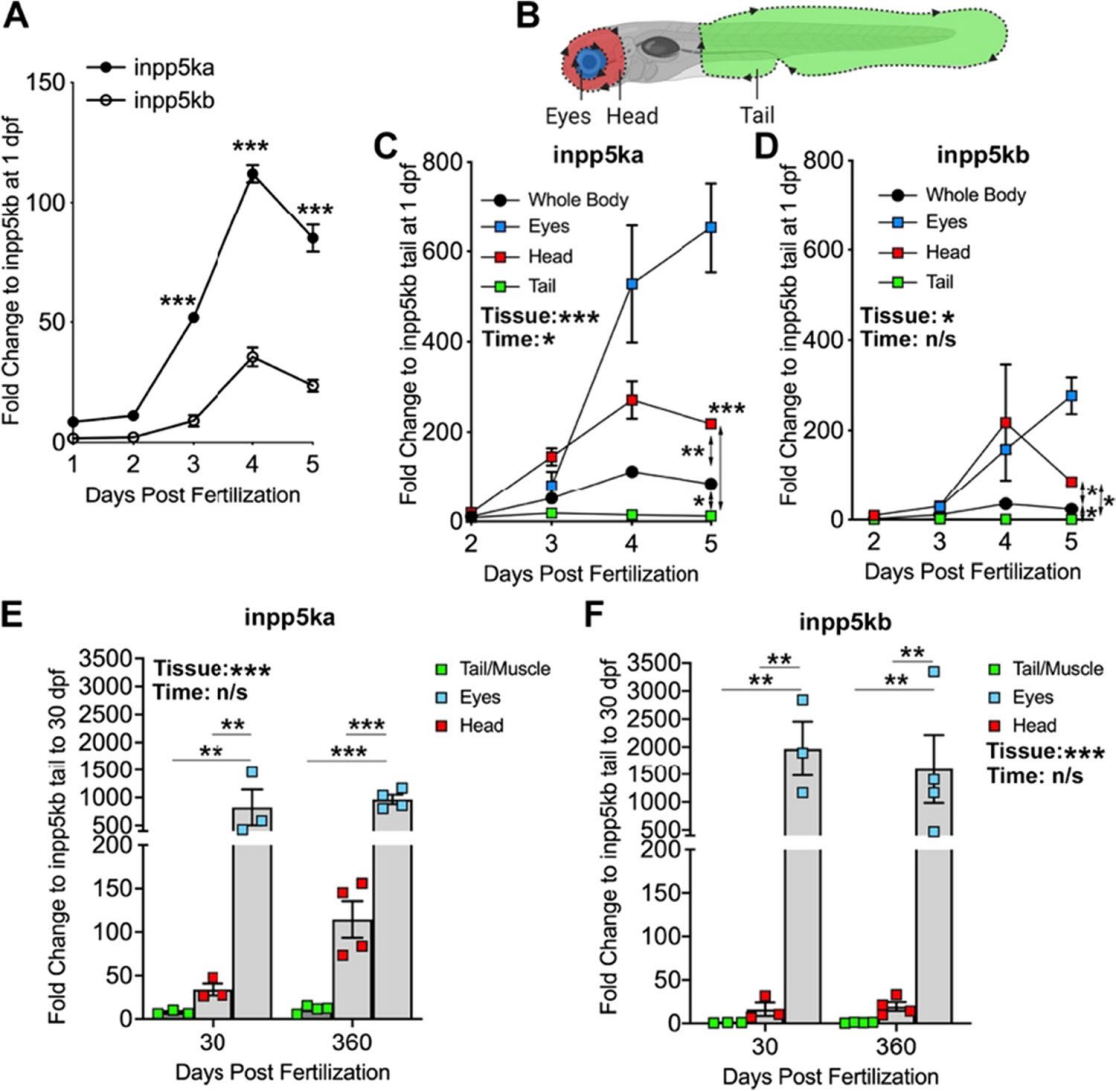
*inpp5ka* and *inpp5kb* mRNAs differ in expression levels in zebrafish larvae. **A** Gene expression determined by qPCR. *inpp5ka* is more highly expressed in whole body lysates through 5 dpf. **B** Larval tissues were excised from the eye, head, and tail for localized gene expression analysis excluding the area of the trunk around the yolk. **C**, **D** Expression for both *inpp5ka* (**C**) and *inpp5kb* (**D**) is low in the tail and increases in the eyes and brain. By 5 dpf, both are most highly expressed in the eyes. Results from the whole body from (**A**) are shown as reference. **E, F** *inpp5ka* (**E**) maintains higher expression levels than *inpp5kb* (**F**) in the tail/muscle and head at 30 dpf and in adult fish. Both genes are expressed at very high levels in the eyes. Values are averages ± SEM. 2-way ANOVA results for tissue and developmental timepoint (time) are also listed. *p < 0.05, **p < 0.01, ***p < 0.001

Analysis of *inpp5ka* and *inpp5kb* mRNA obtained from whole zebrafish embryos had shown higher expression of *inpp5ka* (Osborn et al. 2017). We used qPCR to quantify expression patterns throughout the first five days of development. We found that *inpp5ka* (NM_001089493.1) was consistently expressed much more abundantly than *inpp5kb* (XM_021479346.1) (Fig. 2A). The developmental expression trend was similar for *inpp5ka* and *inpp5kb.* For comparison across the two genes fold changes for the whole embryo were quantified compared to *inpp5kb* expression at 1 dpf since it showed the lowest levels. Both genes showed relatively low levels at 1 and 2 dpf, but expression increased after 3 dpf and we saw 5.8, 3.2, and 3.6 higher expression of *inpp5ka* at 3, 4, and 5 dpf, respectively (Fig. 2A, fold change calculated to 1 dpf *inpp5kb*. *inpp5ka*: 1 dpf 8.5 ± 0.8, 2 dpf 11.1 ± 1.5, 3 dpf 51.9 ± 0.4, 4 dpf 112.1 ± 3.6, 5 dpf 84.9 ± 5.7; *inpp5kb*: 2 dpf 2.1 ± 0.1, 3 dpf 9.0 ± 2.3, 4 dpf 35.5 ± 3.9, 5 dpf 23.6 ± 2.5. *p* > 0.0001 at 3, 4, and 5 dpf).

Loss of *INPP5K* in humans affects the muscle, brain, and eyes and knockdown of *inpp5ka* in zebrafish larvae resulted in morphological abnormalities in the eyes and skeletal muscle (Osborn et al. 2017; Wiessner et al. 2017; Hathazi et al. 2021). We dissected the heads, eyes, and tails of developing larvae for tissue-specific expression analysis (Fig. 2B). Tissue-specific analysis revealed that, while *inpp5ka* was consistently expressed at higher levels than *inpp5kb*, both paralogs exhibit the greatest expression in the eyes, intermediate expression in the head, and low levels in the tail. For comparison, we calculated fold changes compared to expression levels of *inpp5kb* at 1 dpf. *inpp5ka* expression increased dramatically in the eyes by 5 dpf (Fig. 2C). Both genes showed similar increases in the head by 4 dpf with tail levels remaining consistently low (Fig. 2C, D, fold change relative to 1 dpf *inpp5kb* in the tail. *inpp5ka*: 3 dpf head 145.2 ± 19.3, eyes 80.4 ± 31.6; 4 dpf head 271.2 ± 41.5, eyes 528.1 ± 129.8; 5 dpf 218.5 ± 2.7, eyes 653.1 ± 99.6. *inpp5kb*: 3 dpf head 30.4 ± 3.4, eyes 28.6 ± 4.4; 4 dpf head 217.3 ± 128.8, eyes 157.7 ± 8.4; 5 dpf head 85.8 ± 7.9, eyes 276.9 ± 40.6). Since expression levels in the developmental time course seemed to be dropping after 4 dpf, we asked whether tissue-specific expression differences were still present in juvenile (30 dpf) and adult (1 year). Both *inpp5ka* and *inpp5kb* showed very high expression levels in the eyes which were comparable at both ages (Fig. 2E, F). mRNA expression in the head was higher for *inpp5ka* and increased in adulthood. While expression in the tail at 30 dpf and in dissected muscle at 1 year was low for *inpp5ka*, but still 7.6 to 11 times higher than *inpp5kb* (Fig. 2E, F, fold change relative to 30 dpf *inpp5kb* tail. *inpp5ka*: 30 dpf tail 7.6 ± 1.4, head 33.9 ± 6.9, eyes 828.9 ± 320.6; 360 dpf tail 11.3 ± 2.1, head 114.5 ± 21.0, eyes 965.9 ± 83.8. *inpp5kb*: 30 dpf head 16.3 ± 7.1, eyes 1967.1 ± 484.7; 360 dpf tail 0.99 ± 0.22, head 19.6 ± 5.1, eyes 1597.9 ± 615.8). Overall, *inpp5ka* showed consistently higher levels of expression than *inpp5kb* throughout the body apart from the eyes where expression of both genes was the highest at juvenile and adult timepoints.

**Fig. 3:**
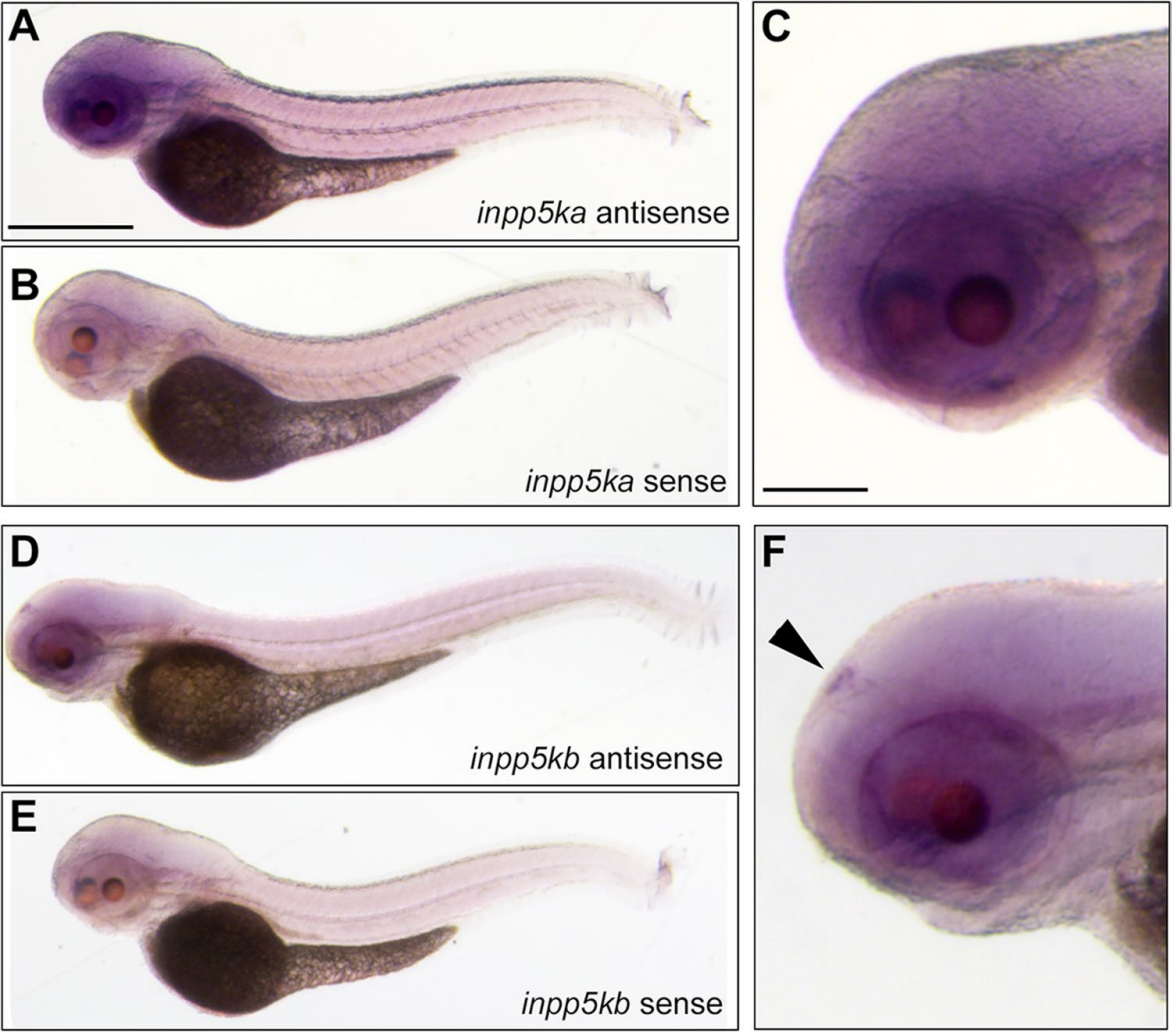
*inpp5ka* and *inpp5kb* mRNAs differ in localization at 3 dpf. **A–C** In situ hybridization in 3 dpf larvae shows that *inpp5ka* mRNA is highly expressed throughout the head and eyes. Scale bars: 500µm in (**A**), 100µm in (**C**). **D–F** *inpp5kb* expression is concentrated to the pineal gland. The pineal gland is indicated by the black arrow

**Fig. 4:**
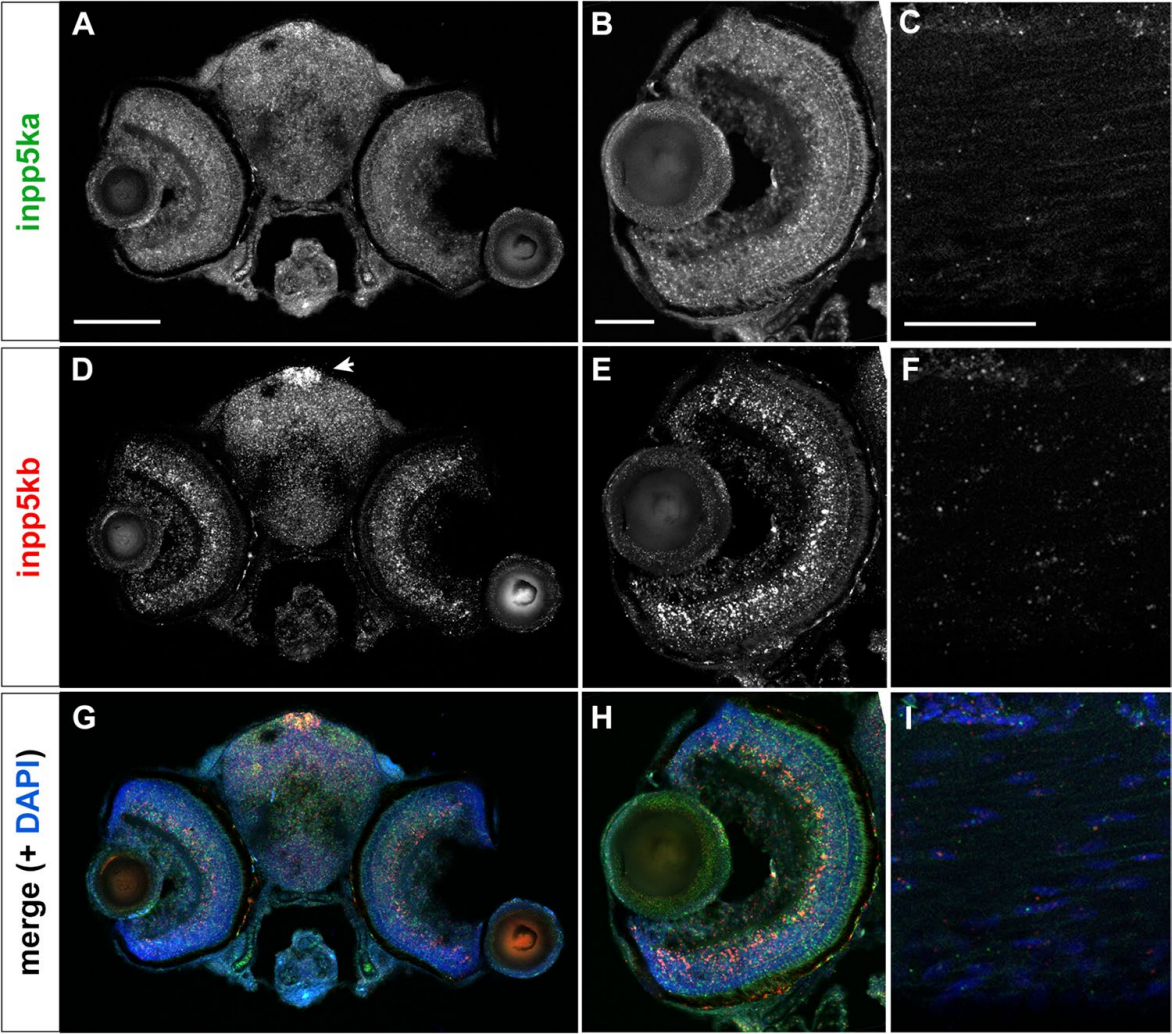
Cellular distribution of *inpp5ka* and *inpp5kb* at 5 dpf by RNA scope in situ. **A–C** *inpp5ka* shows even distribution in the brain and eye (**A**, **B**), also labeling lens cells (**B**). Muscle expression is sparse and at lower levels (**C**). **D–F** *inpp5kb* brain expression is highest in the pineal gland (arrow in **D**). Increased expression is also noted in the inner nuclear layer of the retina with lower expression in photoreceptors, retinal ganglion cells, and lens cells (**E**, also see Suppl. Figure 1B). Sparse expression is present in muscle cells (**F**, also see Suppl Fig. 1C). **G–I** Merged images including both probes (*inpp5ka* in green and *inpp5kb* in red) and DAPI to counterstain the nuclei. Scale bars: 100 µm for (**A**), (**D**), and (**G**), 50 µm for all other panels

To confirm the expression patterns, we first conducted in situ hybridization on whole-mount larvae at 3 dpf when expression begins to diverge. *inpp5ka* antisense probes reflected the results of qPCR expression assays. *inpp5ka* mRNA was most abundant in the head and eyes, with lower expression in the tail (Fig. 3A–C). As expected, *inpp5kb* antisense targeting revealed lower expression throughout the head and eyes (Fig. 3D, E). However, in contrast with *inpp5ka*, *inpp5kb* was abundantly expressed in the pineal gland (Fig. 3F), a neuroendocrine organ which responds to light and plays a role in circadian rhythm (Cahill 1996; Vatine et al. 2011; Livne et al. 2016). These findings suggested that in addition to lower expression, *inpp5kb* may have also diverged in its expression pattern. To better define the cellular distribution of the two mRNAs we then performed fluorescent RNA scope in situ on tissue cryosections at 5 dpf when expression levels are significantly increased via qPCR. This analysis revealed additional differences. *inpp5ka* was evenly distributed throughout the brain, in all layers of the retina, and was also present around the nuclei of lens cells (Fig. 4A, B). Expression in the muscle at the same exposure was much lower reflecting qPCR results (Fig. 4C). *inpp5kb* showed striking differences in distribution with very high expression in the pineal gland (Fig. 4D, Suppl.Fig. 1A) and in the inner nuclear layer of the retina (Fig. 4E, Suppl. Fig. 1B), though expression was still noted in the brain, other retinal layers, and the lens. As seen for *inpp5ka*, *inpp5kb* expression in the muscle was very low (Fig. 4F) though nuclei are sparse in this tissue and staining is less concentrated than in the brain and eyes (Suppl.Fig. 1C). Thus, the mRNA expression levels of *inpp5kb* in the brain and eye appear to be primarily driven by specific expression patterns while *inpp5ka* has a more even distribution pattern.

### Divergence in phosphatase activity of human and zebrafish orthologs of INPP5K

**Fig. 5:**
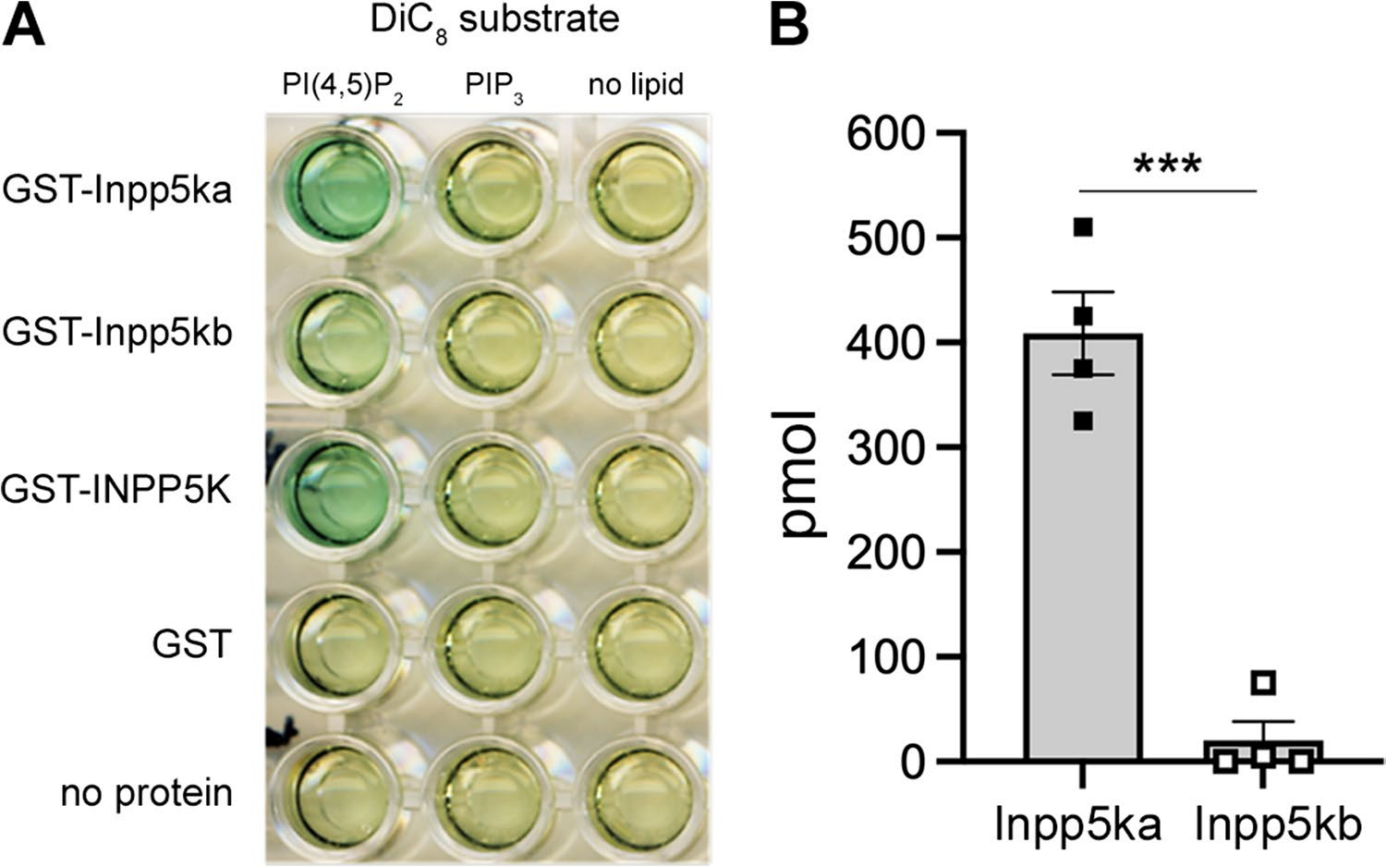
Inpp5ka and Inpp5kb exhibit different phosphatase activity. **A** Phosphatase activity of human INPP5K, Inpp5ka, and Inpp5kb in the malachite assay. Human INPP5K and Inpp5ka demonstrate high activity for the PI(4,5)P_2_ substrate. PIP_3_ did not elicit activity from any isoform. **B** Inpp5ka is more significantly active against diC8PI(4,5)P_2_ compared to Inpp5kb. Values are averages ± SEM. ***p < 0.001 following a *t*-test

To evaluate the preservation of the PI phosphatase activity in the zebrafish isoforms, we conducted a malachite phosphatase assay to examine the activity of INPP5K and the two zebrafish Inpp5k isoforms against PIP_3_ and the preferred substrate PtdIns(4,5)P_2_ (Fig. 5A). We found that zebrafish Inpp5ka and human INPP5K were both highly active against PtsIns(4,5)P_2_ as expected. This activity was specific, as illustrated by the lack of phosphatase activity against PIP_3_. However, compared to Inpp5ka, Inpp5kb was nearly inactive against PtdIns(4,5)P_2_. Inpp5ka yielded 409 pmol of free phosphate vs 20 pmol for Inpp5kb, indicating that Inpp5ka had a 20-fold higher activity compared to Inpp5kb (Fig. 5B).

The INPP5K protein is primarily composed of a 5-phosphatase domain between amino acids 16–318 and a SKITCH domain between amino acids 321–448. Most mutations identified in humans are missense and have been shown to reduce or ablate phosphatase activity (Osborn et al. 2017; Wiessner et al. 2017). We wondered whether the loss in activity in Inpp5kb could be caused by changes in amino acids identified to be critical for the catalytic activity of INPP5K. Basing this analysis on the available crystal structures of other Type II inositol phosphate 5-phosphatases, *INPP5B* and *SYNJ1* (Trésaugues et al. 2014; Paesmans et al. 2020), we found that all sites were conserved in Inpp5ka and Inpp5kb and there were no major changes that could explain differences in activity (asterisks in Fig. 1). We also assessed whether residues known to be affected by pathogenic variants in patients were conserved in Inpp5kb, and these amino acids were all maintained (small arrowheads in Fig. 1) (D’Amico et al. 2020; Osborn et al. 2017; Hathazi et al. 2021; Wiessner et al. 2017; Yousaf et al. 2017). Thus, possible changes in known residues do not explain the difference in function between Inpp5ka and Inpp5kb.

## Discussion

*INPP5K* mutations in humans cause a distinct neurodevelopmental syndrome with variable presentation of intellectual disability, cataracts, short stature, and muscle disease (Osborn et al. 2017; Wiessner et al. 2017; Yousaf et al. 2017; D’Amico et al. 2020). Multiple zebrafish models have been developed to study *inpp5ka/b* function using morpholino oligonucleotides either blocking translation or knocking down mRNA expression (Osborn et al. 2017; Wiessner et al. 2017; Hathazi et al. 2021). However, the presence of duplicated *inpp5k* genes, *inpp5ka* and *inpp5kb*, in zebrafish complicates the development of both candidate loss-of-function or point mutations zebrafish models since both orthologs may need to be targeted. Initial functional data from our previous studies had shown that *inpp5ka* knockdown alone was sufficient to replicate the findings in the double gene knockdown (Osborn et al. 2017). In this study, we show that *inpp5ka* and *inpp5kb* have diverged in expression levels, patterns, and function following teleost whole genome duplication (WGD). *inpp5ka*, rather than *inpp5kb*, maintains a higher sequence identity and similar expression pattern to human *INPP5K*, suggesting that genetic removal of this gene may be sufficient to recapitulate the human mutation.

Polyploidization by WGD is a significant driver of evolution (Postlethwait et al. 1998; Sémon and Wolfe 2007). During the period of re-diploidization that follows a WGD event, most redundant genes are eliminated via genomic rearrangements and mutations causing one duplicated copy to become a pseudogene. However, a duplicated gene may be preserved and gradually diverge in expression patterns and/or function during evolution leading to gene adaptation through sub-functionalization or neo-functionalization (Sémon and Wolfe 2007; Kassahn et al. 2009). While *inpp5ka* is broadly and highly expressed throughout the zebrafish larvae, *inpp5kb* is significantly less expressed. Interestingly, both genes show the highest expression in the eye which is maintained to adulthood. *inpp5ka* is evenly distributed in retinal layers during development, matching the *Inpp5k* expression patterns identified in single-cell RNA sequencing (scRNAseq) datasets generated for the chick, and embryonic murine retina and the adult human retina (Lukowski et al. 2019; Balasubramanian et al. 2021; Yamagata et al. 2021). Considering that congenital cataracts are one of the most consistent phenotypes linked to *INPP5K* mutations in humans (Osborn et al. 2017; Wiessner et al. 2017; Yousaf et al. 2017) the high expression of *inpp5ka* in lens cells found in the RNA scope experiments is also of note.

In contrast, we found that *inpp5kb* is highly enriched in the inner nuclear layer closer where amacrine cells and bipolar cells, both interneurons modulating the transmission of visual signals, are located (Connaughton et al. 2004; Zhao et al. 2009). Additional differential expression was found in the brain revealing widespread and sustained expression of *inpp5ka* while *inpp5kb* is highly enriched in the pineal gland. The pineal gland is thought to be the master regulator for circadian rhythm in vertebrates. Melatonin is the key circadian hormone secreted by the pineal gland in zebrafish (Cahill 1996) and is thought to play a role in locomotor activity (Livne et al. 2016), as well in the timing of reproduction and feeding (Piccinetti et al. 2013). It will be interesting in the future to determine the role of Inpp5kb in pineal and retinal functions independently of its phosphatase activity. This divergence in expression patterns further supports sub-functionalization of *inpp5kb*.

Additionally, we found that Inpp5kb exhibits minimal phosphatase activity against the traditional substrate of INPP5K, PtdIns(4,5)P_2_ (Ijuin et al. 2000; Vandeput et al. 2006). In humans, much of the pathology resulting from mutations within *INPP5K* have been attributed to the dysregulation of phosphoinositide homeostasis (Osborn et al. 2017; Wiessner et al. 2017; McGrath et al. 2020; Hathazi et al. 2021). Most known mutations in INPP5K are missense variants occurring in the catalytic phosphatase domain reducing or ablating conversion of PtdIns(4,5)P_2_ to PtdIns(4)P (Osborn et al. 2017; Wiessner et al. 2017). In the muscle, INPP5K is involved in insulin signaling through the PI3K/Akt/mTOR pathway (Ijuin and Takenawa 2015; Ijuin et al. 2015), but studies in a muscle-specific *Inpp5k* mouse knock-out line also determined that abnormal accumulation of PtdIns(4,5)P_2_ led to a severe disruption in lysosome recycling (McGrath et al. 2020). Interestingly, lysosome enlargement and autophagy inhibition found in the *Inpp5k*-deficient muscle were not dependent of Akt/mTOR signaling, suggesting an independent additional role for PtdIns(4,5)P_2_ in muscle maintenance in the autophagic lysosome reformation pathway (McGrath et al. 2020). In addition, increased levels of D3-phosphoglycerate dehydrogenase (PHGDH) have been found in fibroblasts obtained from individuals with INPP5K phosphatase mutations, indicating further metabolic disruptions (Hathazi et al. 2021). It remains unclear whether INPP5K has additional functions independent of its phosphatase activity. It has been involved in endoplasmic reticulum (ER) organization by multiple groups despite the absence of its substrates in the ER (Dong et al. 2018; Ramos et al. 2019). It is recruited there via interactions with the ER transmembrane protein ARL6IP1 but it is not known whether it is acting on neighboring membranes or performing other functions as part of a complex (Dong et al. 2018). Studies on the different functions and interactions of wild-type and phosphatase-dead zebrafish Inpp5ka and of Inpp5kb and their different isoforms may help shed light on the diverse subcellular activities of these proteins.

Overall, we propose that targeting the phosphatase domain in Inpp5ka would lead to a reliable model for *INPP5K* mutations in humans. Whether Inpp5kb evolved to perform a different function in specific subsets of cells and how it lost its phosphatase activity in the zebrafish remains to be studied.

## Supplementary Information

Below is the link to the electronic supplementary material.

Supplementary file1 (PDF 235 KB)

## Data Availability

All data generated or analyzed during this study are included in this published article.
