# Supplementary material for: Duplicated zebrafish (Danio rerio) inositol phosphatases inpp5ka and inpp5kb diverged in expression pattern and function
Source: Dev Genes Evol. 2023 May 15;233(1):25–34. doi: 10.1007/s00427-023-00703-z (PMC10239392; doi:10.1007/s00427-023-00703-z)
Supplement: Supplementary file 1 — Supplementary file1 (PDF 235 KB) [file 427_2023_703_MOESM1_ESM.pdf]

Supplementary materials

Duplicated zebrafish (*Danio rerio*) inositol phosphatases *inpp5ka* and *inpp5kb* diverged in expression pattern and function

Dhyanam Shukla[1*], Brian M. Gural[1*], Edmund S. Cauley[2*], Namarata Battula[1], Shorbon Mowla[1], Brittany F. Karas[1], Llion E. Roberts[3], Luca Cavallo[1], Luka Turkalj[1], Sally A. Moody[4], Laura E. Swan[3], M. Chiara Manzini[1]

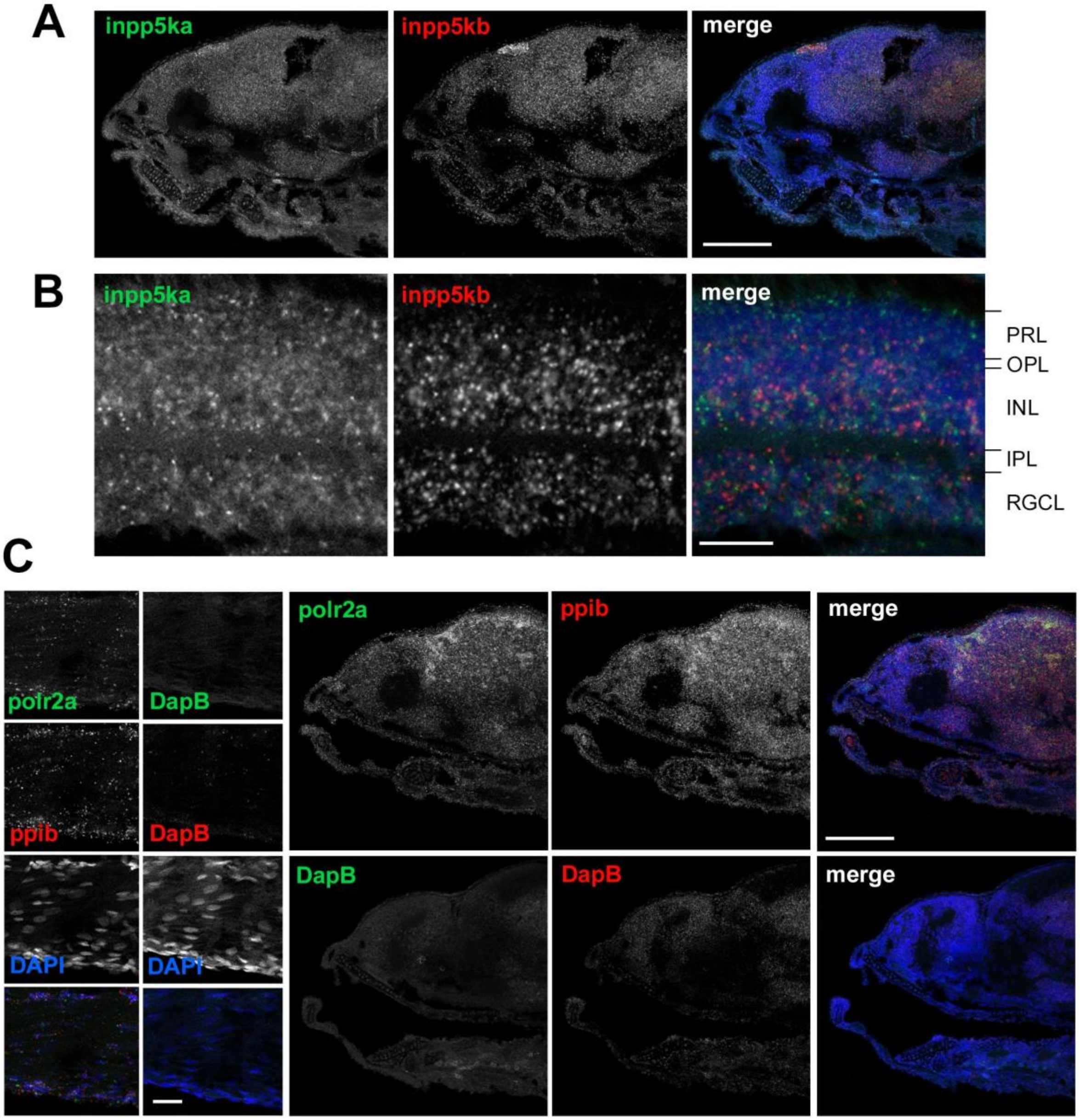

Supplementary Figure 1. Additional data and controls for RNA scope in situ in Figure 4. A. Sagittal section of the 5 dpf zebrafish brain showing broad distribution of both *inpp5ka* and *inpp5kb* and enrichment of *inpp5kb* in the pineal gland. Scale bar: 100μm. **B.** Higher magnification image of the zebrafish retina showing distribution of *inpp5ka* and *inpp5kb* in retinal layers. *inpp5kb* shows the highest expression in the

inner nuclear layer (INL). PRL: photoreceptor layer, OPL: outer plexiform layer, INL: inner nuclear layer, IPL: inner plexiform layer, RGCL: retinal ganglion cell layer. Scale bar: 25μm. **C.** Positive control probes show the expected ubiquitous distribution for *polr2a* and *ppib*. Negative controls taken at the same magnification and exposure show very low background staining from a bacterial probe (DapB). A similar muscle section to the one shown in **Figure 4C**, **F** and **I** is shown on the left for both positive and negative controls. Since nuclei are sparsely distributed in muscle tissue, the staining granules are less concentrated than in the brain, but still show ubiquitous expression. Sagittal brain section at a similar location to **A.** are shown on the right. Scale bar for muscle: 25μm. Scale bar for brain: 100μm.
